# Porcine Circovirus 2 Induction of ROS Is Responsible for Mitophagy in PK-15 Cells via Activation of Drp1 Phosphorylation

**DOI:** 10.3390/v12030289

**Published:** 2020-03-06

**Authors:** Yikai Zhang, Renjie Sun, Xiaoliang Li, Weihuan Fang

**Affiliations:** Zhejiang Provincial Key Laboratory of Preventive Veterinary Medicine, Institute of Preventive Veterinary Medicine, Zhejiang University, Hangzhou 310058, China; 11517030@zju.edu.cn (Y.Z.); rjsun@zju.edu.cn (R.S.)

**Keywords:** porcine circovirus 2, ROS, mitophagy, Drp1

## Abstract

Mitochondrial dynamics is essential for the maintenance of cell homeostasis. Previous studies have shown that porcine circovirus 2 (PCV2) infection decreases the mitochondrial membrane potential and causes the elevation of reactive oxygen species (ROS), which may ultimately lead to mitochondrial apoptosis. However, whether PCV2 induce mitophagy remains unknown. Here we show that PCV2-induced mitophagy in PK-15 cells via Drp1 phosphorylation and PINK1/Parkin activation. PCV2 infection enhanced the phosphorylation of Drp1 and its subsequent translocation to mitochondria. PCV2-induced Drp1 phosphorylation could be suppressed by specific CDK1 inhibitor RO-3306, suggesting CDK1 as its possible upstream molecule. PCV2 infection increased the amount of ROS, up-regulated PINK1 expression, and stimulated recruitment of Parkin to mitochondria. N-acetyl-L-cysteine (NAC) markedly decreased PCV2-induced ROS, down-regulated Drp1 phosphorylation, and lessened PINK1 expression and mitochondrial accumulation of Parkin. Inhibition of Drp1 by mitochondrial division inhibitor-1 Mdivi-1 or RNA silencing not only resulted in the reduction of ROS and PINK1, improved mitochondrial mass and mitochondrial membrane potential, and decreased mitochondrial translocation of Parkin, but also led to reduced apoptotic responses. Together, our study shows that ROS induction due to PCV2 infection is responsible for the activation of Drp1 and the subsequent mitophagic and mitochondrial apoptotic responses.

## 1. Introduction

Porcine circovirus 2 (PCV2) is the major pathogen of several diseases, which are collectively referred to as Porcine-Circovirus-Related Diseases (PCVAD) [1]. As a single-stranded circular DNA virus of only approximately 1.7 kb, PCV2 contains three major open reading frames (ORFs): ORF1 coding for the replicase protein (Rep), ORF2 for the capsid protein (Cap), and ORF3 for a putative protein with pro-apoptotic activity [2,3]. Our recent work has revealed that PCV2 infection induces mitochondrial apoptosis independent of ORF3 [4]. We have also found that PCV2 infection elicits autophagy via activation of CaMKKβ/AMPK/mTOR [5,6]. 

Autophagy is an intracellular event that degrades surplus cytoplasmic components [7,8]. There are three different patterns of autophagy: microautophagy [9], chaperone-mediated autophagy [10], and macroautophagy [11]. Organelle-specific selective autophagy of the mitochondria (mitophagy) is a part of the macroautophagy that plays an important role in the quality control of mitochondria and the maintenance of cell homeostasis via selective engulfment of damaged mitochondria by autophagosomes and their subsequent degradation by lysosomes [12]. Mitochondria are highly dynamic organelles that constantly divide and fuse. Mitochondrial fission is regulated by cytoplasmic dynamic relative protein 1 (Drp1), which is recruited to the surface of mitochondria after its phosphorylation at Ser616 [13] under the control of cyclin B/cyclin-dependent kinase 1 (CDK1) [14]. The PTEN-induced putative kinase 1 (PINK1)-Parkin (E3 ubiquitin ligase) pathway is one of the well-studied multiple molecular regulatory mechanisms in mitophagy [15]. Through the rapid degradation of mitochondrial proteases, PINK1 is maintained at a low level [16]. However, during mitochondrial stress, PINK1 stabilizes on the outer membrane of mitochondria and recruits cytosolic Parkin to the mitochondria [16,17]. Once transferred to the mitochondrial surface, Parkin mediates the engulfment and subsequent destruction of mitochondria through autophagosomes [18]. 

As mitochondria function as the central executioner in the mitochondrial apoptotic pathway, it is possible that mitochondrial dynamics related proteins might participate in the modulation of mitophagy and mitochondrial apoptosis. It has been proved that the Hepatitis B virus (HBV) [19] and the Hepatitis C virus (HCV) [20] could stimulate mitophagy. Much evidence suggests that ROS promotes mitochondrial dysfunction and apoptosis [21,22]. PCV2 infection is known to induce oxidative stress in PK-15 cells [23], autophagy [24], and even mitochondrial apoptosis via the elevation of cellular ROS and cytosolic Ca^2+^ [4,6]. However, whether PCV2 induces mitophagy and whether mitophagy and mitochondrial apoptosis are associated with mitochondrial fission remains to be studied. 

We attempted to examine the effects and mechanisms of PCV2 infection on the mitophagic and mitochondrial apoptotic responses in PK-15 cells. Our data reveal that ROS induced by PCV2 infection elicits Drp1 phosphorylation and activation of the PINK1/Parkin pathway with subsequent mitophagy and mitochondrial apoptosis. 

## 2. Materials and Methods 

### 2.1. Cell Culture and Virus

The porcine kidney cell line (PK-15) free of PCV1 was used. The cells were cultured at 37 °C with 5% CO_2_ in Dulbecco’s Modified Eagle’s Medium (DMEM) (HyClone, South Logan, UT, USA) containing 4% heat-inactivated fetal bovine serum (GIBCO, Grand Island, NY, USA) with 1% L-glutamine, 1% non-essential amino acids, 100 U/mL penicillin G, and 100 μg/mL streptomycin (100×, GIBCO). The PK-15 cells that stably expressed EGFP-LC3 (PK-15/EGFP-LC3) were generated and stored in our laboratory. 

The PCV2 strain YW (GenBank accession no. MG245866) belonged to PCV2b and was originally isolated from the lung of a pig with naturally occurring PCVAD in Zhejiang Province, China. The virus was propagated in PK-15 cells and was used to generate the isogenic mutant with inactivation of the ORF3 protein (ΔORF3) [4]. The PCV2 parent strain YW and its ΔORF3 mutant contained 1×10^5.25^ and 1×10^5.1^ infectious virus particles per mL, respectively. As PCV2b has been the predominant genotype in the swine population and had a more significant effect than the PCV2d in inducing mitochondrial apoptosis [4], this genotype was chosen in this study to investigate its role in mitophagy.

### 2.2. Chemicals and Antibodies

N-acetyl-L-cysteine (NAC) (Beyotime, Hangzhou, China); Mdivi-1 (MedChemExpress, Monmouth Junction, NJ, USA), a selective cell-permeable inhibitor of mitochondrial division; and RO-3306 (Selleck Chemicals LLC, Houston, TX, USA), a selective CDK1 inhibitor were used in their respective experiments. Antibodies against β-actin, caspase 3A/B, PINK1, LC3Ⅰ/Ⅱ, GADPH, CDK1 (cdc2), and Drp1 and its phosphorylated form (p-Drp1) were purchased from Cell Signaling Technology (CST, Boston, USA). Anti-VDAC1 antibody was from Abcam and the Anti-Parkin antibody was from Proteintech (Chicago, USA). Mouse anti-Cap monoclonal antibody was produced in our laboratory. Goat anti-rabbit or goat anti-mouse antibodies conjugated to horseradish peroxidase (HRP) were purchased from KPL (Gaithersburg, MD, USA).

### 2.3. Virus Infection and Chemical Treatments

PK-15 cells at approximately 50–60% confluence were infected with PCV2 or its ΔORF3 mutant strain at a multiplicity of infection (MOI) of 1 for 48 h to examine mitophagy, oxidative stress, or apoptosis. To investigate the effects of the oxidative stress and mitochondrial fission inhibitors on the changes of ROS and relevant molecules, PK-15 cells were first infected with PCV2 and then treated with NAC (10 mM) or Mdivi-1 (4 μM) at 2 h post infection (hpi). The cells were subjected to further incubation for specified periods according to the experimental goals. Mock-infected cells were included as controls. All infected and/or treated cells were collected for relevant assays described below. 

### 2.4. Protein Extraction and SDS-PAGE/Western Blotting

PK-15 cells cultured in 24-well plates were washed with phosphate-buffered saline (PBS at pH 7.2, Beyotime) and collected in a cell lysis buffer (Beyotime) containing protease inhibitors (Roche Molecular Biochemicals, Mannheim, Germany) on ice. The cell lysates were centrifuged at 14,000× *g* for 10 min at 4 °C and the supernatant samples were collected. To isolate the mitochondrial fraction, PK-15 cells were infected with PCV2. At 48 hpi, the cytosolic and mitochondrial fractions were isolated using a Mitochondria Isolation Kit (Beyotime) according to the manufacturer’s recommendations. The protein concentration was determined using a Bradford assay kit (Beyotime).

Equal amounts of protein samples were loaded and separated on 8%, 10%, or 15% SDS–PAGE gels. The samples were transferred to polyvinylidene fluoride (PVDF) membranes (Merck Millipore, Darmstadt, Germany). After blocking for 1 h with PBS with 0.05% Tween-20 (PBST) containing 5% nonfat milk power at 37 °C, the membranes were incubated with primary antibodies at 4 °C overnight. The membranes were then washed with PBST and incubated with secondary antibodies at 37 °C for 1 h. Images of the immunoblots were captured using a Gel 3100 chemiluminescent imaging system (Sagecreation, Beijing, China). The target protein blots were quantified using the NIH ImageJ software (National Institutes of Health, Germany). 

### 2.5. Detection of Mitophagy

Mitophagy Detection Kit^®^ (Dojindo, Kumamoto, Japan) containing Mtphagy Dye^®^ and Lyso Dye^®^ was used to detect the mitophagy induced by the PCV2 infection. PK-15 cells were incubated in 24-well plates and stained with 100 nM Mtphagy Dye for 15 min in the dark before washing with DMEM (HyClone). The cells were then infected with PCV2 (MOI = 1). At 48 hpi, the infected cells were washed with DMEM and Lyso Dye (1 μM) was added into each well. The plate was incubated for another 10 min, followed by washing with Hanks’ balanced salt solution (HBSS) (Beyotime). The cells were imaged on a confocal fluorescence microscope (IX81-FV1000, Olympus, Markham, ON, Canada). The fluorescence intensity was quantified using the NIH ImageJ software.

The colocalization of mitochondria autophagosomes was detected using confocal microscopy (IX81-FV1000). PK-15/EGFP-LC3 cells and MitoTracker™ Red CMXRos (ThermoFisher, Waltham, USA) labeling were used to examine the colocalization of mitochondria with GFP-LC3-positive autophagosomal structures. 

### 2.6. Transmission Electron Microscopy

Transmission electron microscopy (TEM) was used to assess the mitochondrial morphology. PK-15 cells were incubated in a six-well plate. Mock- and PCV2-infected cells (48 hpi) were prepared for the TEM. The specimens were first fixed with 2.5% glutaraldehyde in a phosphate buffer (PB) (0.1 M, pH 7.0) for 4–5 h, and then washed three times with PB for 15 min at each step. The specimens were postfixed with 1% OsO_4_ in PB for 1–2 h and washed three times with PB as per above. Dehydration of the specimens was conducted using a graded series of ethanol (30%, 50%, 70%, 80%, 90%, 95%, and 100%) for about 15 to 20 min at each step, and then using absolute acetone for 20 min. For embedding and ultrathin sectioning, specimens were placed in Eppendorf tubes containing Spurr resin and heated at 70 °C for at least 9 h. The LEICA EM UC7 ultratome (Wetzlar, Germany) was used to prepare the sections, which were stained with uranyl acetate and alkaline lead citrate for 5 to 10 min, and then observed using the Hitachi Model H-7650 TEM (Tokyo, Japan).

### 2.7. Immunofluorescence

PK-15 cells were cultured in Petri dishes (10 mm in diameter) (Xinyou, Hangzhou, China) and infected with PCV2 at MOI = 1. Mock-infected cells were included as a control. At 48 hpi, MitoTracker™ Red CMXRos (ThermoFisher) was used to stain the mitochondria for 15 min at 37 °C. The cells were fixed with 4% paraformaldehyde for 30 min, washed twice with HBSS, and then permeabilized with HBSS containing 0.25% TritonX-100 for 15 min. The cells were then probed with the Anti-p-Drp1 antibody. Images were visualized under a 60× oil objective on a confocal microscope (IX81-FV1000, Olympus). The quantification of the cell fluorescence intensity was conducted with the NIH ImageJ software.

### 2.8. Measurement of the Mitochondrial Mass, ROS, and Mitochondrial Membrane Potential

For the determination of mitochondrial mass, ROS, and mitochondrial membrane potential (MMP), PK-15 cells were cultured in 24-well plates. Briefly, after removing the culture medium, cells were washed with HBSS. The mitochondrial mass was probed using 0.1 μM MitoTracker Green (excitation—490 nm; emission—516 nm) (Dojindo), ROS levels were probed using 10 μM of 2′,7′-dichlorofluorescein diacetate (DCFH-DA) (excitation—480 nm; emission—525 nm) (Sigma-Aldrich, St. Louis, MO, USA), and MMP was probed using 0.1 μM JC-1 (excitation—490 nm; the monomeric form: emission—527 nm; J-aggregates: emission—590 nm.) (BD Biosciences, San Diego, CA, USA) for 30 min at 37 °C in the dark following the manufacturer’s instructions. After staining, the cells were trypsinized, collected using centrifugation (3 min, 500× *g*), and re-suspended in HBSS for flow cytometric analysis. All flow cytometric experiments were performed on the BD FACSCalibur™ Flow Cytometer (BD Biosciences, San Diego, CA, USA). Data were analyzed using FlowJo software, version 10 (Treestar, San Carlos, CA, USA). 

### 2.9. Measurement of Apoptosis

For the determination of the apoptotic rate, an Annexin V-FITC Apoptosis Detection Kit (Vazyme, Nanjing, China) was used for the flow cytometry. After 48 hpi, PK-15 cells were trypsinized and collected in HBSS, where 400 μL 1× binding buffer was added to re-suspended cells for each sample. The cells were stained with 5 μL Annexin V-FITC and 5 μL propidium iodide (PI) for 10 min in the dark at room temperature. At least 1 × 10^4^ cells in each sample were measured to identify the apoptotic cell populations. 

To detect whether virus infection activated caspase 3 and caspase 9, the Caspase 3/9 Activity Assay Kit (Beyotime) was used. The kit contains Ac-DEHD-pNA and Ac-LEHD-pNA as caspase 3 and 9 specific substrates, respectively. The active caspases could cleave substrates to become fluorescent (excitation at 485 nm and emission at 530 nm). 

### 2.10. Statistical Analysis

The results are expressed as mean ± SEM from at least three independent experiments. Statistical comparisons were made using one-way analysis of variance (ANOVA) followed by a least significant difference (LSD) post hoc test. Differences between groups were considered significant if * *p* < 0.05 and highly significant if ** *p* < 0.01. 

## 3. Results

### 3.1. PCV2 Induced Mitophagy with the Accumulation of Mitophagosomes

**Figure 1:**
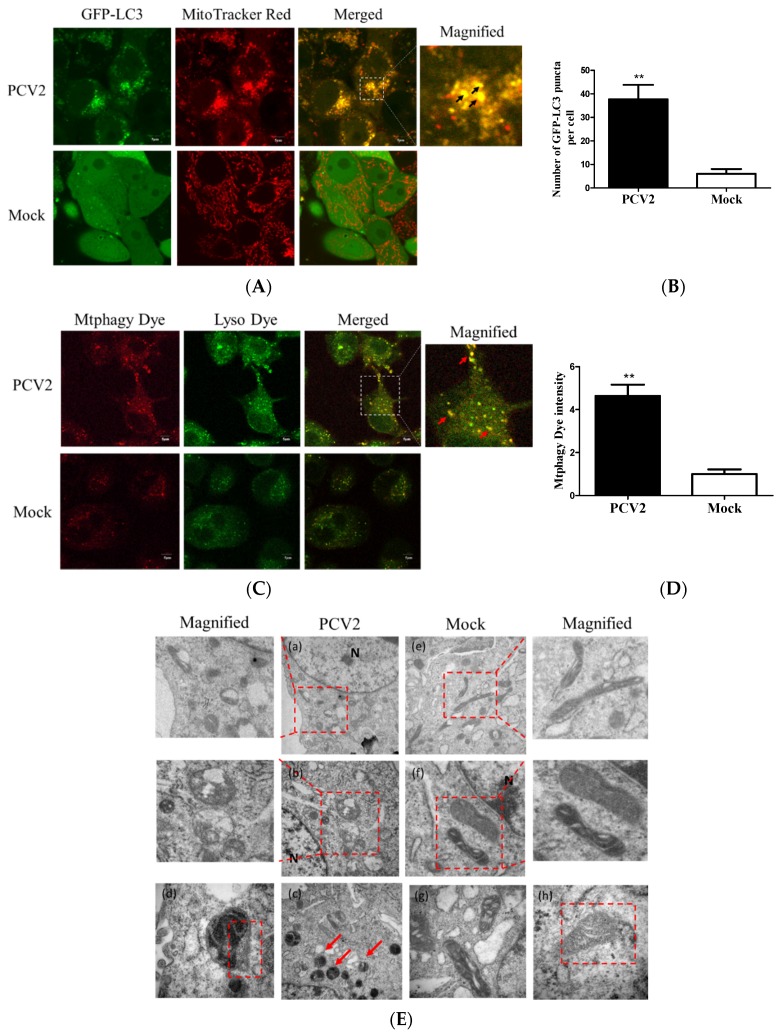
The PCV2-induced formation of mitophagosomes. PK-15 cells stably expressing EGFP-LC3 with PCV2 infection (MOI = 1) or without (Mock) at 48 hpi were examined for the formation of mitophagosomes. (**A**) The cells were stained with MitoTracker Red. Images on the right depict the magnified fields of the boxed areas in the merge images, showing colocalization of GFP-LC3 punctae (green) with mitochondria (Red). Images in the white boxes were enlarged 2.5-fold. Scale bars: 5 μm. The black arrows indicate the colocalization of mitochondria and LC3 dots. (**B**) PCV2 infection induced a higher number of mitophagosomes in mitochondria compared to the mock control. (**C**) The cells were stained with Mtphagy Dye and Lyso Dye. Images on the right depict the magnified fields of the boxed areas in the merge images. Images in the white boxes were enlarged 2.5-fold. Scale bars: 5 μm. The Mtphagy punctae (red) and Lyso puncta (green) were colocalized (red arrows), indicating the occurrence of mitochondrial autophagy (mitophagy). (**D**) PCV2 infection induced a higher intensity of Mtphagy Dye than the mock control. (**E**) Transmission electron microscopy images revealed the mitochondrial ultrastructure in PCV2-infected or mock-infected cells. In the magnified images, the red dotted boxes indicate swollen mitochondria (**a**,**b**) and an autophagic vacuole that contains mitochondria (**c**,**d**). In the mock-infected cells, the normal mitochondria were seen as clear cristae and filling matrix (**e**–**h**). The magnified images were enlarged twofold. Scale bars: 200 nm. Bar charts (**B**,**D**) show the mean ± SEM of three independent experiments. ** *p* < 0.01.

A previous work in our lab has shown that PCV2 could induce autophagy in PK-15 cells [24]. To examine whether PCV2 could induce mitophagy, PK-15/EGFP-LC3 cells were infected with PCV2 for the confocal microscopy analysis of mitophagosome formation by merging GFP-LC3 puncta with MitoTracker Red. A significantly higher number of EGFP-LC3 puncta were seen in the mitochondria in PCV2-infected cells compared to the uninfected cells (** *p* < 0.01) (Figure 1A,B). Mtphagy Dye exhibited a higher fluorescence intensity upon fusion between the mitochondria and lysosomes as an indication of mitophagy. Figure 1C shows that the mitophagic vacuoles and lysosomes were colocalized in PCV2-infected PK-15 cells. The fluorescent intensity was higher in PCV2-infected cells than mock-infected ones (** *p* < 0.01) (Figure 1D). TEM images revealed mitochondrial swelling and mitochondria lacking cristae in PCV2-infected cells (Figure 1Ea–c). PCV2 infection induced the appearance of autophagic vacuoles (Figure 1Ed), suggesting the occurrence of autophagy of damaged mitochondria. In the mock-infected cells, mitochondria with intact cristae could be readily observed (Figure 1Ee–h). These data suggest that the PCV2 infection induced mitochondrial damage and might induce the selected elimination of damaged mitochondria via autophagy.

### 3.2. PCV2 Enhanced Drp1 Ser616 Phosphorylation and Its Mitochondrial Translocation

**Figure 2:**
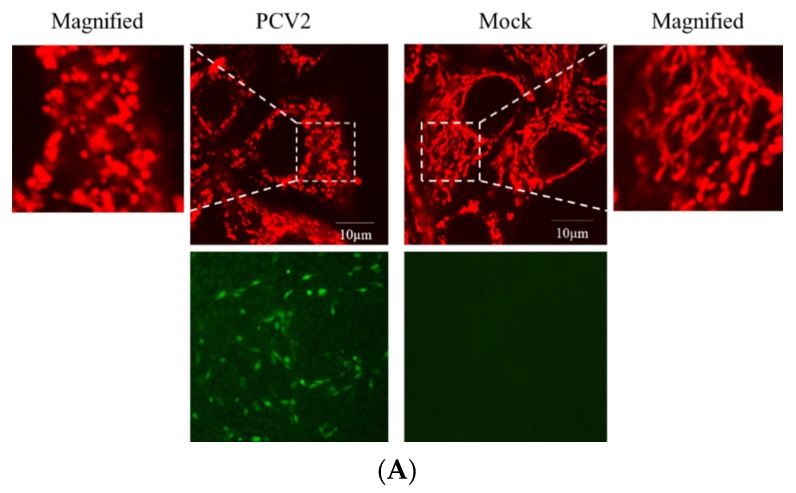

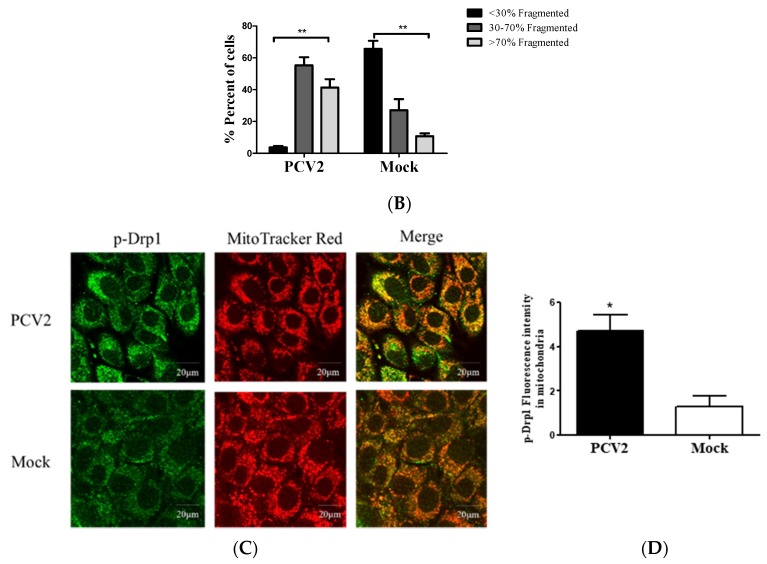
Defects in the mitochondrial tubular networks and mitochondrial translocation of the mitochondrial-fission-related protein Drp1 induced by PCV2. PK-15 cells were infected with PCV2 for 48 h in Petri dishes for confocal microscopy. Mock-infected cells were used as a control. (**A**) Confocal microscopy images showing mitochondrial fragmentation in PCV2-infected cells. At 48 hpi, the cells were stained with MitoTracker (red) (top panels) or immunostained with anti-PCV2 capsid protein antibody (green) (bottom panel). In the magnified images, typical tubular mitochondria in mock-infected cells and fragmented mitochondria in infected cells are shown. (**B**) Quantification of the mitochondrial morphology in (A). Cells were scored as one of the three morphological categories as depicted in the inset. In each group, at least 50 cells were scored. (**C**) The cells were prestained with MitoTracker Red (100 nM) and then immune-stained with anti-phospho-Drp1 (Ser616) (green) at 48 hpi. (**D**) The quantification of the p-Drp1 (Ser616) fluorescence intensity of (**C**) was analyzed using NIH ImageJ software in at least 50 cells of each group. Bar charts (**B**,**D**) show the mean ± SEM of three independent experiments. * *p* < 0.05 and ** *p* < 0.01.

Viruses, such as HBV [19], HCV [20], and the classical swine fever virus (CSFV) [25], could perturb mitochondrial dynamics by causing mitochondrial fission and mitophagy. However, it is unclear whether abnormal mitochondrial dynamics occur in cells infected with PCV2. Confocal immunofluorescence was performed to observe the changes in mitochondrial dynamics in PK-15 cells infected by PCV2. Figure 2A,B shows that PCV2 infection shifted the morphology of mitochondrial networks toward a significantly higher proportion of fragmented structures (higher proportion of >70% fragmented than the proportion of <30% fragmented structures in the PCV2 infected cells, ** *p* < 0.01). In uninfected cells, however, the proportion of <30% fragmented structures was dominant. To explore whether PCV2 induced Drp1 translocation to mitochondria, PCV2-infected cells were analyzed using confocal microscopy with a specific antibody that recognizes the Ser616-phosphorylated Drp1 (p-Drp1). We found a significant elevation of p-Drp1 intensity in PCV2-infected cells with its translocation to mitochondria compared with the mock cells (Figure 2C,D), indicating that PCV2 promoted Drp1 phosphorylation and its recruitment to mitochondria.

**Figure 3:**
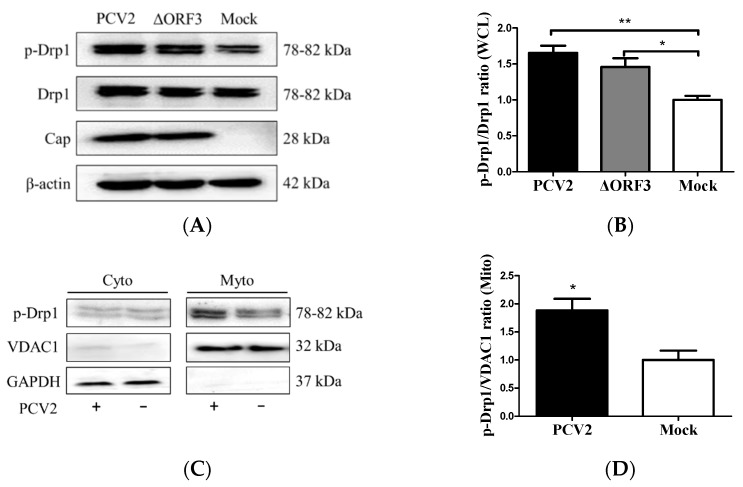
PCV2 stimulated Drp1 phosphorylation and its translocation to mitochondria independent of ORF3. The cells were infected with PCV2 or its mutant ΔORF3 (MOI = 1) in 24-well plates for 48 h. Mock-infected cells were used as a control. (**A**) Whole-cell lysates (WCL) extracted from PK-15 cells infected with PCV2 and ΔORF3 infection were analyzed using Western blotting with antibodies against Drp1, p-Drp1 (Ser616), Cap (PCV2 capsid protein), and β-actin (used as a protein loading control). (**B**) The ratio of p-Drp1/Drp1 in whole-cell lysates (WCL) is shown. (**C**) Cytosolic (Cyto) and mitochondrial (Mito) fractions isolated from PK-15 cells with or without PCV2 infection were analyzed using Western blotting with p-Drp1, VDAC1, and GAPDH antibodies. GADPH was used as the internal control of cytoplasmic proteins and VDAC1 as that of mitochondrial proteins. (**D**) The ratio of p-Drp1/VDAC1 in the mitochondrial fraction is shown. Bar charts (**B**,**D**) show the mean ± SEM of three independent experiments. * *p* < 0.05 and ** *p* < 0.01.

The ORF3 protein was found to cause apoptosis in PCV2-infected cells [2]. We hypothesized that PCV2-induced mitochondrial fission might occur independently of ORF3. An ORF3-inactivated PCV2 mutant (ΔORF3) was used to infect the PK-15 cells. In the whole-cell lysate, there was increased p-Drp1 in PCV2-infected cells compared with the control cells (** *p* < 0.01). The ORF3-deficient virus could still induce Drp1 phosphorylation (* *p* < 0.05), although the responses were generally lower than those observed with the parental strain (Figure 3A,B). To further substantiate the observation that PCV2 induced Drp1 translocation to mitochondria, we extracted mitochondria from virus-infected and mock-infected PK-15 cells for Western blotting. We found that p-Drp1 was significantly enriched in the mitochondrial fraction of PCV2-infected cells (* *p* < 0.05) (Figure 3C,D). These results indicated that PCV2 promoted mitochondrial fission via Drp1 phosphorylation and its mitochondrial translocation.

### 3.3. PCV2-Induced ROS was Involved in Drp1 Phosphorylation

**Figure 4:**
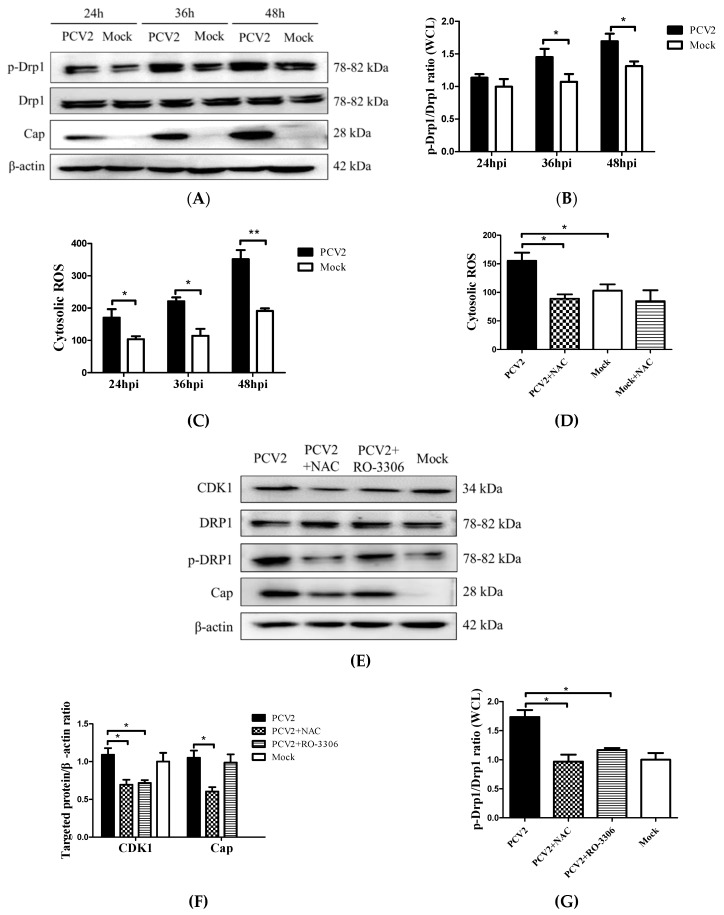
PCV2 infection promoted the Drp1 phosphorylation and reactive oxygen species (ROS) generation, and Drp1 activation could be reversed via treatment with N-acetyl-L-cysteine (NAC) or via the inhibition of CDK1 with RO-3306. PK-15 cells were infected with PCV2 (MOI = 1) for 24, 36, or 48 h. Mock-infected cells were used as a control. (**A**) Target proteins of the PK-15 cell extracts were separated using SDS-PAGE and analyzed using Western blotting with antibodies to p-Drp1 (Ser616), Drp1, and PCV2-Cap. β-actin was used as a protein loading control. The gels shown are representative of three individual experiments. (**B**) The p-Drp1/Drp1 ratio in WCL. (**C**) After 48 hpi, cells were harvested to assess cytosolic ROS using flow cytometry. Cells were treated with 10 μM DCFH-DA for 30 min at 37 °C in the dark. The cytosolic ROS levels are shown as the mean value of DCF fluorescence intensity. (**D**) The PK-15 cells were infected with PCV2, treated with 10 mM NAC at 2 hpi, and then incubated for an additional 46 h. The cells were collected for flow cytometry to examine the changes in the cytosolic ROS level. (**E**) The cells were infected with PCV2, treated with 10 mM NAC or 200 nM RO-3306 at 2 hpi, and then incubated for an additional 46 h before measurement of the target protein expression using Western blotting for CDK1, Drp1, p-Drp1 (Ser616), and PCV2-Cap. β-actin was used as a protein loading control. The gels shown are representative of three individual experiments. (**F**) Ratios of CDK1 and PCV2-Cap to β-actin. (**G**) The p-Drp1/Drp1 ratio in WCL. Data in bar charts (**B**–**D**,**F**,**G**) show the mean ± SEM of three independent experiments. * *p* < 0.05 and ** *p* < 0.01.

We previously found that the PCV2 infection of PK-15 cells caused a significant elevation of cellular ROS [4]. To investigate the relationship between increased ROS levels and Drp1 phosphorylation induced by PCV2, we collected cells at different time points from 24 hpi to 48 hpi for Western blotting and flow cytometry. Figure 4 shows that there was a significant increase of both p-Drp1 and ROS with the progression of the PCV2 infection, particularly after 36 hpi (Figure 4A–C). NAC treatment was effective at suppressing the ROS generation in PCV2-infected cells (Figure 4D) and at inhibiting Drp1 phosphorylation, suggesting that ROS was involved in the Drp1 phosphorylation (Figure 4E,G). The cyclin B/CDK1 complex is one of the mitotic kinases that is known to cooperate with the small G protein RALA and its effector RALBP1 to promote Drp1 phosphorylation and mitochondrial fission [26,27]. A specific CDK1 inhibitor RO-3306 was used to treat PCV2-infected cells to examine whether CDK1 regulated the phosphorylation of Drp1 during the PCV2 infection. We found that the suppression of CDK1 by RO-3306 inhibited the Drp1 phosphorylation induced by PCV2 (* *p* < 0.05), while the NAC treatment decreased the CDK1 expression (* *p* < 0.05) (Figure 4F). These findings suggest that CDK1 might be the upstream signal of Drp1 phosphorylation in response to ROS.

### 3.4. PCV2-Induced Drp1 Phosphorylation and Mitophagy Could be Reversed Using ROS Scavenging N-acetyl-L-cysteine

**Figure 5:**
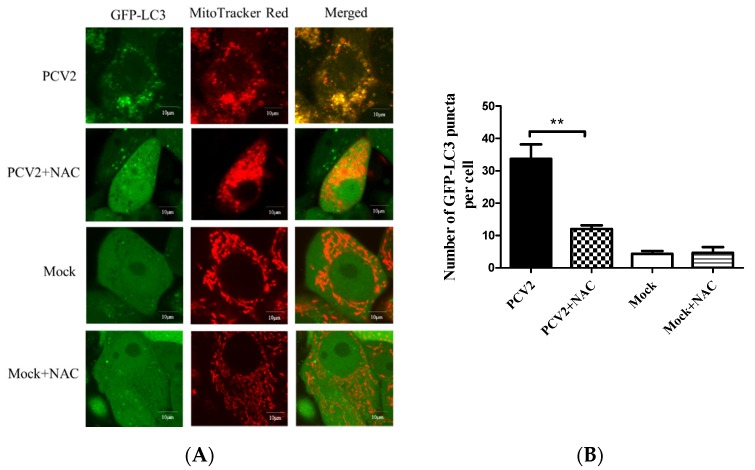
Colocalization of the EGFP-LC3 punctae and MitoTracker induced by PCV2 was inhibitable using a ROS-scavenger N-acetyl-L-cysteine (NAC). The PK-15 cells stably expressing EGFP-LC3 in Petri dishes were infected with PCV2, treated with 10 mM NAC at 2 hpi or untreated, and subjected to further incubation for 46 h. (**A**) Cells were treated with 100 nM MitoTracker Red for 15 min in the dark. Representative confocal images showing the colocalization of MitoTracker Red and EGFP-LC3 punctae in the cells with or without (Mock) PCV2 infection. Scale bars: 10 μm. (**B**) Average numbers of colocalization of EGFP-LC3 and MitoTracker Red punctae per cell from at least 50 cells. Bar chart (**B**) shows the mean ± SEM of three independent experiments. ** *p* < 0.01.

Mitochondrial function is accompanied by ROS generation. Excessive ROS generated by mitochondrial dysfunction might induce mitophagy, a mechanism used to eliminate the damaged organelles [28]. PCV2 infection is known to elevate the ROS load [4,23,29]. ROS-scavenging NAC was employed to treat the PCV2-infected cells to investigate the effect of PCV2-induced ROS on mitophagosome formation shown as colocalization of the GFP-LC3 puncta with mitochondria. Figure 5 reveals that NAC treatment significantly reduced mitophagosome formation in PCV2-infected cells (** *p* < 0.01).

**Figure 6:**
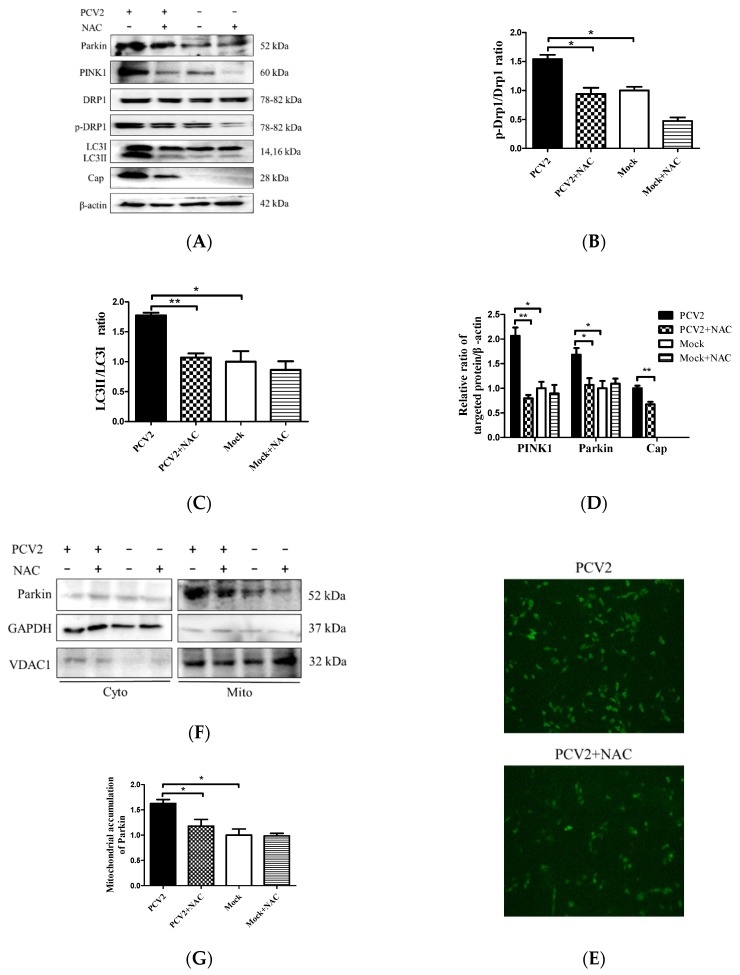
PCV2 increased the Drp1 phosphorylation, LC3II conversion, and PINK1 and Parkin expression, which could be down-regulated using an N-acetyl-L-cysteine (NAC) treatment. The PK-15 cells were infected with PCV2, treated with 10 mM NAC at 2 hpi, and then incubated for an additional 46 h before measuring the target protein expression using Western blotting or immunofluorescence. (**A**) Whole-cell extracts were separated using SDS-PAGE and analyzed using Western blotting with antibodies against Parkin, PINK1, Drp1, p-Drp1 (Ser616), LC3Ⅰ/Ⅱ, Cap, and β-actin. β-actin was used as a protein loading control. The gels shown here are representative of three individual experiments. (**B**–**D**) Ratio of p-Drp1 to Drp1 (**B**); LC3Ⅱ/LC3Ⅰ(**C**); and PINK1, Parkin, and Cap to β-actin (**D**). (**E**) The PCV2-infected cells with or without NAC treatment were immunostained with an anti-PCV2-Cap monoclonal antibody. (**F**) Parkin in cytoplasmic (Cyto) and mitochondrial (Mito) fractions was visualized using Western blotting. GAPDH was used as the internal control for the cytoplasmic proteins and VDAC1 for that of the mitochondrial proteins. (**G**) Ratio of Parkin to VDAC1 in the mitochondrial fraction. Bar charts (**B**–**D**,**G**) show the mean ± SEM of three independent experiments. * *p* < 0.05 and ** *p* < 0.01.

To further address how ROS were involved in the Drp1 phosphorylation and PCV2-induced mitophagy, we examined the changes of PINK1, Parkin, LC3II, Drp1, and p-Drp1 in whole lysates of cells infected with PCV2 with or without NAC treatment. We observed that PCV2 infection led not only to autophagy, shown as a marked increase of the LC3II/LC3I ratio, but also to the elevated expression of PINK1 and Parkin in PK-15 cells, as well as increased Drp1 phosphorylation, which are indicative of mitophagic responses. All these changes due to PCV2 infection were reversed using an NAC treatment (Figure 6A–D) (* *p* < 0.05 or ** *p* < 0.01). PCV2 replication was also inhibited using an NAC treatment, which is shown as a decreased PCV2 Cap expression and a reduced number of PCV2 infected cells (Figure 6D,E). Further analysis revealed that increased Parkin accumulation in the mitochondrial fraction of PCV2 infected cells could be blocked using an NAC treatment (* *p* < 0.05) (Figure 6F,G). Thus, we provide clear evidence that there was a causal link between mitophagy and excessive ROS production during PCV2 infection, and that ROS might function as an upstream signal for the activation of Drp1 phosphorylation with subsequent mitochondrial fission and mitophagy. 

### 3.5. Suppression of the Mitochondrial Fission Protein Drp1 Inhibited PCV2-Induced Mitophagy and Mitochondrial Apoptosis

**Figure 7:**
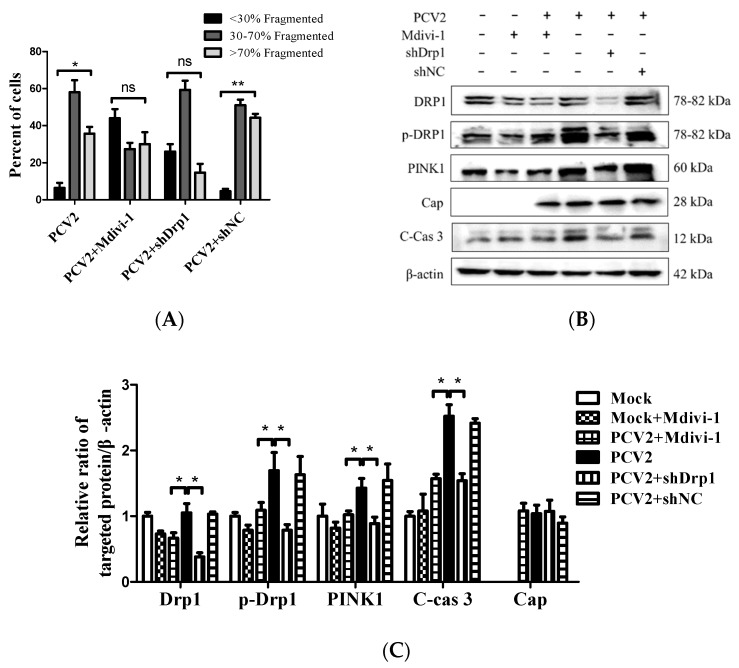

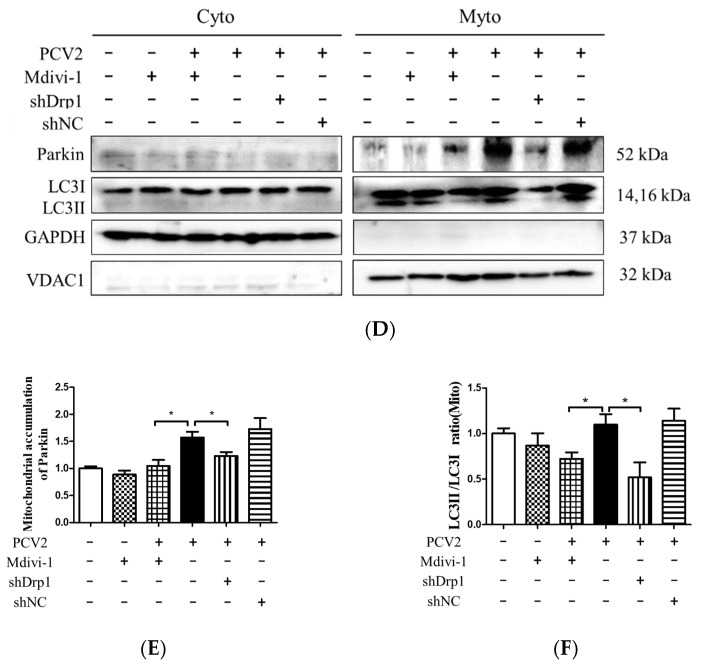
Inhibition of Drp1 by shDrp1 and Mdivi-1 suppressed mitochondrial fragmentation, Drp1 phosphorylation, and PINK1 expression, as well as reduced the accumulation of Parkin and LC3II in mitochondrial fraction and caspase 3 cleavage. PK-15 cells were incubated in 24-well plates. Cells were either infected with the lentivirus containing shDrp1 (shDrp1) for 12 h and then infected with PCV2 or the cells were infected with PCV2 and then treated with 4 μM Mdivi-1 at 2 hpi, and then incubated for an additional 46 h before confocal microscopy analysis or measuring the target proteins using Western blotting. (**A**) Quantification of mitochondrial fragmentation in PCV2-infected cells. (**B**) Whole-cell extracts were separated using SDS-PAGE and analyzed using Western blotting with antibodies against Drp1, p-Drp1 (Ser616), PINK1, PCV2 Cap, cleaved caspase 3 (C-cas 3), and β-actin. β-actin was used as a protein loading control. The results shown here are representative of three individual experiments. (**C**) The ratios of Drp1, p-Drp1 (Ser616), PINK1, C-cas 3, and Cap to β-actin. (**D**) The levels of Parkin and LC3II in the cytoplasmic (Cyto) and mitochondrial (Mito) fractions were analyzed using Western blotting. GAPDH was used as the internal control of cytoplasmic proteins and VDAC1 as that of the mitochondrial proteins. (**E**) Ratio of Parkin to VDAC1 in the mitochondrial fraction. (**F**) Ratio of LC3Ⅱ/Ⅰ in the mitochondrial fraction. Bar charts (**A**,**C**,**E**,**F**) show the mean ± SEM of three independent experiments. * *p* < 0.05 and ** *p* < 0.01.

Mitochondrial dynamics is regulated by Drp1, a key protein involved in mitochondrial fission [30], and integrally linked to apoptosis [31]. We showed that PCV2 could induce mitochondrial apoptosis [4]. To gain insight into the role of Drp1 in PCV2-induced mitophagy and mitochondrial apoptosis, we tested whether the specific suppression of Drp1 with lentivirus-mediated shRNA (shDrp1) or chemical treatment with Mdivi-1 would affect the mitophagic and apoptotic responses. The chemical inhibition of Drp1 or RNA silencing with shDrp1 could effectively alleviate mitochondrial fragmentation caused by PCV2 infection (Figure 7A). To confirm the relationship between Drp1 and mitophagy, Western blotting was used to examine the effects of Drp1 inhibition on the expression of key molecules involved in mitophagy and apoptosis. We found that the inhibition of Drp1 expression resulted in decreased PINK1 and caspase 3 cleavage (Figure 7B,C), as well as a diminished Parkin accumulation and reduced LC3II/I ratio in the mitochondrial fraction (Figure 7D,E)(* *p* < 0.05 compared with those of the cells infected with PCV2 only).

**Figure 8:**
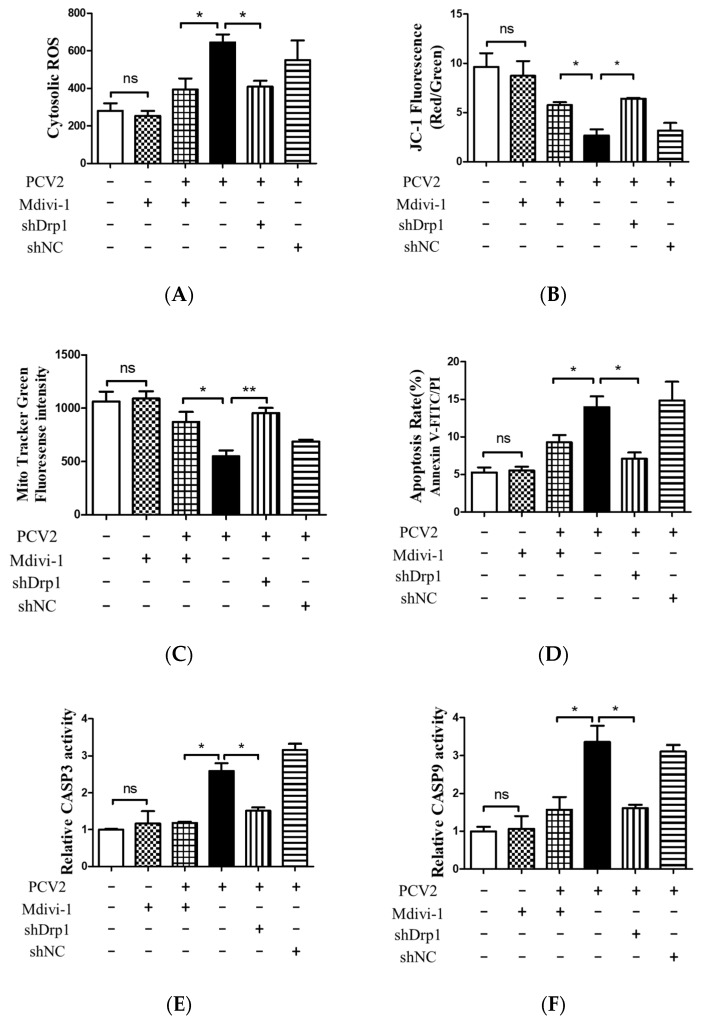
Inhibition of Drp1 by shDrp1 and Mdivi-1 decreased the cytosolic ROS, rescued mitochondrial mass and MMP, and reduced apoptosis due to the infection by PCV2. PK-15 cells in 24-well plates were first infected with the lentivirus containing specific shDrp1 (shDrp1) for 12 h and then infected with PCV2 for 48 h. Alternatively, PK-15 cells were infected with PCV2, treated with 4 μM Mdivi-1 at 2 hpi, and then incubated for an additional 46 h. (**A**) Cytosolic ROS in the cells were evaluated using flow cytometry, shown as the mean value of the DCF fluorescence intensity. (**B**) JC-1 fluorescence was depicted as the ratio between red and green fluorescent signals to indicate changes in the mitochondrial membrane potential. (**C**) Mitochondrial mass was measured with MitoTracker green staining using flow cytometry. (**D**) Apoptosis rates of PK-15 cells examined using flow cytometry. (**E**,**F**) The bar charts show fold changes of caspase 3 (**E**) and caspase 9 (**F**) enzyme activities in the cells. Bar charts (**A**,**C**,**E**,**F**) show the mean ± SEM of three independent experiments. * *p* < 0.05 and ** *p* < 0.01.

The down-regulation of Drp1 by Mdivi-1 treatment or shDrp1 significantly decreased ROS and rescued MMP in PCV2-infected cells compared with PCV2-infected but untreated cells (* *p* < 0.05) (Figure 8A,B). Drp1 suppression inhibited the loss of mitochondrial mass in cells infected by PCV2 (* *p* < 0.05, ** *p* < 0.01 vs. control cells) (Figure 8C). Treatment with either Mdivi-1 or shDrp1 could alleviate apoptotic responses in PK-15 cells, which was shown as a decreased apoptotic rate (* *p* < 0.05) (Figure 8D) and reduced activities of caspases 3 and 9 that were closely related to mitochondrial apoptosis (* *p* < 0.05) (Figure 8E,F). These findings indicate that Drp1 might function as an upstream molecule and cooperate with ROS to regulate the PINK1/Parkin pathway, as well as mitophagic and mitochondrial apoptotic responses.

## 4. Discussion

**Figure 9:**
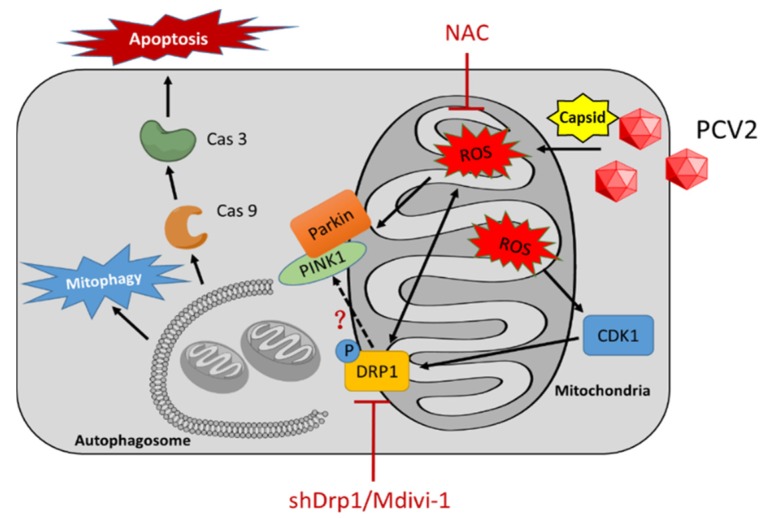
A proposed model of mitophagy and mitochondrial apoptosis induced by PCV2 infection. PCV2 infection induces oxidative stress, loss of mitochondrial mass, and mitochondrial membrane potential, probably through its capsid protein [4], leading to mitochondrial fission, mitophagy, and mitochondrial apoptosis. ROS induced by PCV2 infection stimulates Drp1 phosphorylation, probably by activating its upstream molecule CDK1. N-acetyl-L-cysteine (NAC) treatment could reverse these PCV2-induced phenotypes, and inhibited Drp1 phosphorylation, PINK1 expression, and Parkin translocation to mitochondria. Inhibition of Drp1 via Mdivi-1 treatment or specific RNA silencing could reduce the amount of ROS and alleviate PCV2-induced mitophagy and mitochondrial apoptosis.

Viruses induce host cell responses, such as the unfolded protein response (UPR) and autophagy, and can hijack UPR and autophagy to promote their replication and infectivity [32,33,34]. Induction of autophagy and UPR via viral infection could lead to the activation of persistent cellular stresses, including oxidative stress, which is involved in the activation or modulation of apoptotic pathways [34,35,36]. PCV2, though the smallest one known to infect mammals, is a virus that is versatile in activating host cell responses, such as ER stress [4,37,38], oxidative stress [4,23,29], autophagy [5,6,24], ORF3-dependent apoptosis [3], and ORF3-independent mitochondrial apoptosis [4]. The major questions here are where these cellular responses are intertwined during PCV2 infection and what are the mechanisms of such a crosstalk. Because PCV2 infection induces increased ROS generation and oxidative stress [23,29], we were particularly interested in understanding what roles the ROS play in mitochondrial dynamics toward autophagy and apoptosis. Here, for the first time, we demonstrated that the generation of ROS during PCV2 infection induced mitophagy in PK-15 cells via the ROS-mediated activation of Drp1 (Figure 9). 

The recruitment of Parkin to mitochondria is considered to be a hallmark of mitophagy [12]. PINK1 is another essential component of mitophagy, which is involved in Parkin’s translocation to damaged mitochondria and its subsequent activation [17]. Our study revealed that PCV2-induced mitophagy was shown as a significant translocation of Parkin to mitochondria and increased levels of PINK1 and LC3II in infected cells, as well as perinuclear accumulation of damaged mitochondria and the subsequent formation of mitophagosomes and mitophagolysosomes. Drp1 is known to mediate the scission of mitochondrial membranes, regulate mitochondrial dynamics, and influence mitochondrial apoptosis and mitophagy [39,40]. We found that PCV2 infection caused a time-dependent increase in p-Drp1 and ROS. The fact that PCV2-induced Drp1 phosphorylation and mitophagic responses could be markedly reduced by the ROS scavenger NAC (Figure 6A,B) indicates that ROS generated during PCV2 infection might be the upstream molecule of Drp1 activation with subsequent mitochondrial fission and mitophagy. 

The link between PINK1 and Parkin is well established. PINK1 stabilizes dysfunctional mitochondria and recruits Parkin from the cytosol to damaged mitochondria [41]. However, the relationship between Drp1 and PINK1/Parkin remains elusive. Drp1 and Parkin were co-recruited to mitochondria in the proximity of PINK1 following mitochondrial depolarization, indicating a spatial coordination between these molecules [42]. Our results suggest that Drp1 might have positive effects on the PINK1/Parkin pathway in PCV2-infected cells because the inhibition of Drp1 by Mdivi-1 or RNA silencing decreased the PINK1 expression and reduced the Parkin recruitment to mitochondria (Figure 7). This is similar to the work of Park et al. who showed that the knockdown of Drp1 significantly blocked the increase of PINK1 and Parkin expression in the mitochondrial fraction of cells treated with carbonyl cyanide m-chlorophenylhydrazone (CCCP) [43]. Xiao et al. showed that ROS was able to trigger PINK1/Parkin-dependent mitophagy in CCCP-treated cells [44]. These findings imply that there is a close relationship between Drp1 and PINK1/Parkin in CCCP-induced mitophagy. 

CDK1 is known to mediate mitochondrial activities in the cell cycle progression and stress response [45]. Kashatus et al. showed that the small Ras-like GTPase RALA could be relocalized upon phosphorylation to the mitochondria, where it concentrated RALBP1 and Drp1. RALBP1 was associated with CDK1, which led to phosphorylation of Drp1 on Ser616 [46]. Another report also revealed the involvement of CDK1 in Drp1 phosphorylation and mitochondrial fission [27]. Here, we showed that the specific inhibition of CDK1 by RO-3306 suppressed Drp1 phosphorylation in PCV2 infected cells, and NAC treatment reduced the level of both CDK1 and p-Drp1 (Figure 4). Thus, we speculate that CDK1 might be involved in Drp1 phosphorylation by ROS during PCV2 infection. However, the specific mechanism regarding how CDK1 is activated remains to be explored. 

Mitochondria are known to be the main source of cellular ROS. Mitochondrial dysfunction may lead to the overproduction of ROS [47]. We found that the silencing of Drp1 by shRNA reduced the cellular ROS level. Similar results have been reported in other cell models. In T lymphocytes, ROS generation is dependent on mitochondrial fission. The inhibition of Drp1 resulted in reduced ROS levels [41]. In a high-glucose-induced cell death model, mitochondrial fragmentation was found as an upstream factor for ROS overproduction, and the inhibition of mitochondrial fission normalized the cellular ROS level [48]. Therefore, the role of Drp1 in PCV2-induced mitophagy seems to be two-sided: one as a receiver of the ROS signal, probably via its upstream molecules, and the other as a stimulator of ROS generation via its regulated mitochondrial fission (Figure 9). 

Mitophagy, as a contrast to apoptosis, could be pro-survival to the host cells, favoring viral propagation and persistence. We found that the inhibition of Drp1 by Mdivi-1 or shDrp1 decreased both the mitophagic and mitochondrial apoptotic responses of PCV2-infected cells. This is different from what was seen in other viruses, such as HBV, HCV, CSFV, porcine reproductive and respiratory syndrome virus (PRRSV), and transmissible gastroenteritis virus (TGEV). In cells infected with these viruses, the induction of mitophagy was found to prevent apoptosis, while the inhibition of mitophagy promoted apoptosis [19,20,26,49,50,51]. This may result from different mechanisms of induction of either mitophagy or apoptosis by different viruses. Zhu et al. reported that TGEV infection induced mitophagy and increased the expression of DJ-1 [51], a molecule that is involved in several signaling pathways, including the control of mitochondrial quality and reaction to oxidative stress [52,53], while the silencing of DJ-1 inhibited mitophagy with reduced ROS, but increased apoptosis in the virus-infected cells [51]. They found marked localization of the nucleocapsid protein N of TGEV in the mitochondrial fraction, while the role of the N protein either in mitophagy or apoptosis was unexplored. Results from this study and our recent work have revealed that an ORF3-inactivated PCV2 mutant could still induce mitophagy and mitochondrial apoptosis [4]. PCV2 Cap was sufficient to induce ROS [4], in addition to its ability to cause an unfolded protein response and apoptosis [38]. Therefore, we tend to believe that it is the general oxidative stress due to excessive generation of ROS during PCV2 infection that targets both mitophagy and mitochondrial apoptosis via Drp1 activation, with mitochondrial fission being the initial step of these cellular events [41]. 

In conclusion, our study shows that PCV2-induced ROS was responsible for mitophagic and/or mitochondrial apoptotic responses through the activation of Drp1. Oxidative stress is one of the major factors in PCV2 pathogenesis. This warrants the use of antioxidant treatments in PCVAD as an adjunctive therapy.
